# High Precision Wide Bandwidth DC Current Transducer Based on the Platiše Flux Sensor

**DOI:** 10.3390/s20154197

**Published:** 2020-07-28

**Authors:** Uroš Platiše, Tomaž Kanalec, Mihael Mohorčič

**Affiliations:** 1 ISOTELpower, Smart Energy, Ltd., Trbovlje, 1420 Zasavska, Slovenia; uros@isotel.org (U.P.); tomaz@isotel.org (T.K.); 2 UrsoLab Lda, 9000-238 Madeira, Portugal; 3 Jožef Stefan International Postgraduate School, Jamova 39, SI-1000 Ljubljana, Slovenia; 4 Jožef Stefan Institute, Jamova 39, SI-1000 Ljubljana, Slovenia

**Keywords:** Platiše Flux Sensor, current-controlled variable reluctance (CCVR), isolated direct-current transducer, direct-current current transformer (DC-CT)

## Abstract

In the last decade, we observed a noticeable increase in direct-current systems (DC), particularly in solar power generation, grid storage systems, and electric mobility. Some of these systems may require high-voltage isolation and peak currents in excess of kA. The existing standard compact and lower cost current sensing solutions hardly ever achieve an overall measurement uncertainty below 1% mainly due to offsets and hysteresis; their typical bandwidth is about 250 kHz, and they may also be noisy. This article presents a new method of isolated DC and AC current measurement based on a single gapless core and the innovative Platiše Flux Sensor. After verification in a mixed-signal simulator, the method was implemented in a functional prototype of a DC current transducer (CT) and thoroughly tested in a reference setup. The performance tests showed a low offset and hysteresis, a bandwidth in the MHz range, low power consumption, and low noise operation. Furthermore, the low current transducer achieved a typical uncertainty of less than 0.2% and a linearity of less than 200 ppm, which indicates an overall superior performance compared to representative comparable CTs based on alternative technologies. In addition to the areas of application mentioned above, the new type of DC-CT can be used for general purpose metering, measurement instrumentation, and high power DC and AC systems.

## 1. Introduction

The demand for high-precision current sensors has seen a dramatic increase in the past decade due to recent developments in a number of application areas. These include: (i) the automotive industry for controlling the ever-increasing number of DC motors in traditional, as well as in electric vehicles [1,2]; (ii) smart grids with distributed renewable energy sources and new types of loads requiring precise power quality and consumption monitoring [3,4]; (iii) microgrids with predominantly DC elements and loads [5]; and (iv) the increasingly ubiquitous Internet of Things (IoT) devices [6] in industrial and residential environments with DC current sensing being one of the basic principles for monitoring and controlling a variety of activities. In addition to these emerging technologies, existing high-power applications, such as railways and high power AC distribution and transmission grids [7], also require accurate measurements of the higher harmonics alongside DC (leakage) currents, which also affect the overall accuracy [8].

A wide range of diverse application areas with differing requirements and tolerances regarding the performance of current sensors has given rise to various methods and types of sensors being developed. These utilize different underlying physical principles, from ohmic resistance (e.g., shunt) and induction (e.g., Rogowski transformer, current transformer), to the magnetic field (e.g., Hall-effect sensors, fluxgate transformer, magneto resistance materials), and the Faraday effect (e.g., fiber-optic current sensors) [9], each with its particular advantages and drawbacks. Applications such as massive solar power plants, battery storage systems, and e-mobility require compact and cost-effective wide bandwidth current sensors with a large nominal measurement range from a few A up to several kA, a wide dynamic range with temporary currents of several orders of magnitude above the nominal average (e.g., e-vehicle acceleration, in-rush currents in battery storage, etc.), and a measurement uncertainty 3σ ranging from 0.01% to 0.1%. In light of these challenges, this investigation focuses on designing a gapless core based on the zero-flux principle to retain and improve the superb performance in terms of the sensitivity, immunity, and DC offsets achieved with the fluxgate principle, also considering the null method with compensation winding to achieve the highest possible linearity and the widest frequency bandwidth. The main challenge of the undertaking was to invent a new type of DC flux sensor for a single lower-cost gapless core and a transducer design.

The main original contribution of this article is a new patented [10] method of measuring magnetic flux inside the closed-loop gapless core described in Section 3, based on the novel concept of current-controlled variable reluctance (CCVR) described in Section 3.2. It combines the Platiše Flux Sensor explained in Section 3.3 with the required control circuitry. The aim of this article, however, is to present the basic principle of the new method and its proof-of-concept working prototype implementations used for initial performance evaluation, leaving any necessary adaptation to the method to ensure compliance with application domain-specific standardization to the final product development and industrialization phase.

This article is divided into six sections. After the Introduction, Section 2 provides a brief overview of some of the most popular current sensing methods and their main drawbacks, which motivated the research work presented in this article. Section 3 outlines the basic principle of the main contribution, the Platiše Flux Sensor. This is followed by a description of the complete proposed direct current-current transducer (DC-CT) in Section 4, along with details on its thorough testing, performance evaluation, and a comparison with other technologies in Section 5. Finally, the conclusions and the outlook of this paper are drawn in Section 6.

## 2. Related Background

The increasing demand for current sensing with a broad spectrum of requirements has resulted in different methods and sensors being developed. The most common method to measure current is by measuring the voltage drop on a shunt. A shunt is a low-ohmic resistor with low temperature drifts and is capable of withstanding higher stresses without being damaged. It can be described as:

(1) $$V\left(i,T\right)=i\cdot R_{25}\left(1+\sum TC_{i}\Delta T^{i}+\Delta \left(i,V,RH,t,g\right)\right)+L\frac{di}{dt}+V_{EMF}\Delta T$$

As shown in Equation (1), the voltage drop on a shunt of the nominal resistance $R_{25}$, which is typically given at a nominal ambient temperature around 25 ${}^{\circ }$C, is affected mainly by the self-heating effects. Even though the resistance is low, the total losses can exceed 50 W power dissipation. The temperature stability is denoted by the $TC_{i}$ vs. the temperature changes ΔT. The long-term stability of the resistance is also affected by various stresses, such as (peak) currents *i*, voltage *V*, humidity RH, aging *t*, and mechanical stresses *g*. Then, there is the undesired noticeable inductance *L*, which may at very low resistances $R_{25}$ limit the bandwidth to as low as 10 kHz (R/L). The last, often neglected, but very important part is a thermal electromotive force (EMF) $V_{EMF}$ that is reflected as an offset voltage error.

Accurate and stable resistors with a low temperature drift are costly: they require sufficient space for cooling with heat sinks, and yet, the measurement itself is not isolated, making the overall system solution unsuitable for high power e-mobility, power generation, and distribution grid applications. A more suitable solution is therefore to create a combination of a system that mirrors and downscales high primary currents through an isolation barrier to secondary lower currents, for which high ohmic, cost-effective, lower power, and compact shunts can be used.

Another popular method for current sensing is based on measuring the magnetic field around the conductor with the well-established Hall sensor. The Hall sensor voltage is proportional to (major contributors):

(2) $$V\left(B,T\right)=S\left(B\right)\cdot B+\beta \Delta T\pm V_{off}$$

The sensitivity S(B) of the sensors themselves (without any pre-amplifiers) varies from a few mV to 100 mV per one Tesla and is linear in the limited range of a magnetic field *B*. The higher the sensitivity, the higher the temperature instability β, which typically ranges from 50 ppm/K to 1000 ppm/K, and equivalently the offset voltage $V_{off}$ in the range from 1 to 10 mT. A typical bandwidth of this method is between 10 and 100 kHz. The current is indirectly measured via a magnetic circuit, which may be:

1. open-air, in which a current passes by the Hall sensor or two Hall sensors operating in differential mode [11], mainly to improve the immunity to external fields. This method is typically used for high currents above 100 A,
2. using a soft magnetic material with an air-gap into which a Hall sensor is placed. This method is suitable in a wider range and offers a higher sensitivity due to the gain of the magnetic circuitry.

The described placement of the Hall sensor refers to a so-called open-loop measurement. The measurement uncertainty includes every error from the Hall sensor and any additional errors caused by the magnetic circuitry. The major drawback of this method in an open-air placement is the higher susceptibility to external magnetic fields. Such systems typically have an accuracy (often referred at extended coverage k=2,⋯,2.6) of about ±2.5%. The immunity is greatly improved by using soft-magnetic cores, which work as magnetic amplifiers; however, the magnetic circuit itself adds an additional offset and hysteresis errors in addition to the Hall sensor errors. The magnetic amplification and correlating sensitivity largely depend on the air-gap length and the soft-core permeability, resulting in a so-called effective permeability $\mu_{e}$:

(3) $$\mu_{e}=\frac{\mu_{r}}{1+\mu_{r}\cdot l_{g}/l_{e}}$$

A typical sensor with an air-gap of $l_{g}=1$ mm sufficiently large to insert a Hall sensor, an effective length $l_{e}=1$ dm, and a relative permeability of $\mu_{r}=5000$ yields an effective relative permeability (amplification) of around 98. The air-gap is relatively large, which means a significantly increased magnetic core permeability, i.e., to $\mu_{r}=50$ k, increases the relative effective permeability $\mu_{e}$ by a negligible amount, only to about 100. The preferred way to increase sensitivity and consequently reduce the magnetic offsets and hysteresis anomalies of the soft-core material is therefore to reduce the air-gap length $l_{g}$.

To compensate for the large temperature dependency and non-linearity and to extend the measurement range of such a current transducer, the Hall sensor can be placed in a closed-loop configuration to serve as a linear null detector. The feedback information in such a setup drives back a secondary winding of $N_{s}$ turns with the current equal to $-I_{p}\frac{N_{p}}{N_{s}}$. Only two main sources of errors now remain:

- the offset of the Hall sensor and
- the offset of the magnetic circuit with hysteresis.

The transducer accuracy can be improved by using alternative sensor solutions, such as:

- a magneto-resistance sensor or
- a flux-gate sensor inserted into a core.

The total accuracy of such a sensor is about 1% within the industrial temperature range [12] and the core itself is the main contributor to the error due to the reduced effective permeability. The secondary compensation winding notably also provides a direct AC path, thus extending the AC bandwidth to at least 250 kHz and higher at small signal amplitudes.

**Figure 1:**
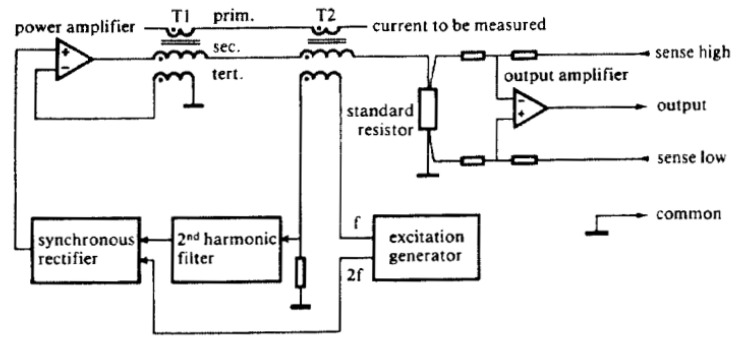
CERN: block diagram of zero-flux DCCT. ©1977 IEEE. Reprinted with permission from [13]. T1 as AC transformer, T2 as zero-flux sensor, prim. as primary current to be measured, and voltage output.

One of the first gapless and wide-bandwidth zero-flux solutions with a block diagram as shown in Figure 1 was introduced at CERN in 1976 [13]. There, the first transformer T1 serves as an AC path, while T2 operates as a zero-flux sensor. The excitation generator is constantly sweeping the current across the entire range from the minimum to the maximum saturation point, thus canceling out the magnetic offsets. If the flux is non-zero, the excitation current becomes asymmetrical, which is easily observable as the second harmonic distortion. It is filtered out and demodulated to drive the compensation power amplifier to compensate the DC offsets. The small signal bandwidth obtained with this solution was 10 kHz, a gain accuracy of 100 ppm, a linearity of 5 ppm, an offset error of only 10 ppm, and temperature drifts of 1 ppm/K.

**Figure 2:**
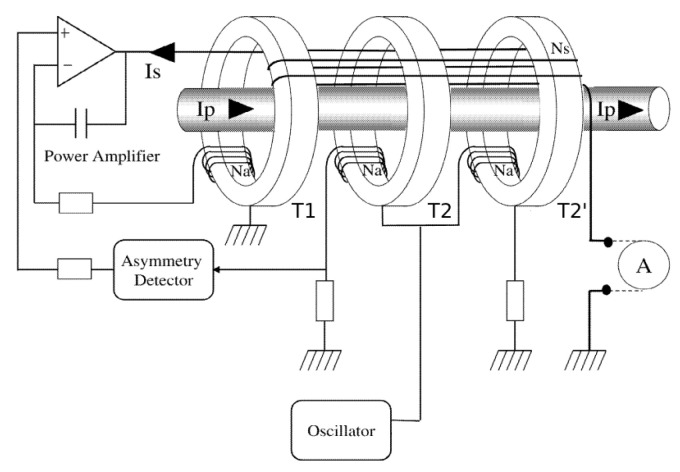
Block diagram of MACC zero-flux DCCT. ©2002 IEEE. Reprinted with permission from [14]. T1 as AC transformer, T2 and T2’ as balanced zero-flux sensor, $I_{p}$ as primary current to be measured, and *A* as current output.

The excitation winding of T2 injects noise into the output, as well as into the primary conductor. The improved version therefore consists of three cores, with the additional T2’ being added to operate in the opposite direction (180${}^{\circ }$ phase shifted), as shown in Figure 2 [14]. When the excitation ampere-turns (At) between T2 and T2’ are in perfect balance, the noise introduced by the excitation generator is canceled out. Sensors based on this zero-flux method are typically bigger-format high-performance instruments that are also power hungry and costly, making them overall unsuitable for mass market use. Products based on this solution include MACC [15], LEM IT [12], and many others.

The same method of flux sensing is used by the more cost-effective so-called standard flux-gate sensors by LEM [12], already mentioned earlier as an alternative to Hall sensors. Their high sensitivity and offset canceling technique have increased the popularity of flux-gate sensors in various configurations, as a magnetic compass or as a general purpose magnetic sensor. Recently, they have even been printed on flex boards [16].

## 3. Platiše Flux Sensor

In order to design an improved DC current sensor, the research was based on a zero-flux gapless core design with a compensation winding, which eventually led to the invention of a patented original magnetic flux sensor named after its inventor, Uroš Platiše [10]. The following section gives an overview of the basic concept of the innovation.

### 3.1. The Concept of Measuring Magnetic Flux

In general, magnetic circuits can be represented with electric circuits, in which the magnetic voltage equals Θ=IN, the magnetic resistance is $\frac{l}{\mu A}$, and the magnetic current ϕ=BA, where *I* is the electric current, *N* is the number of turns, *l* is the effective length, *A* the cross-section, and $\mu =\mu \left(f\right)=\mu_{r}\left(f\right)\mu_{0}=\mu_{r}\left(f\right)\cdot 4\pi 10^{-7}\;(H/m)$, where *f* is a frequency to represent frequency dependent soft-magnetic materials. However, no known element has been discovered to date that could directly measure a constant magnetic voltage drop on a magnetic resistance. The only way to observe a magnetic current, or flux, is therefore to make it non-constant by changing its amplitude or direction.

**Figure 3:**
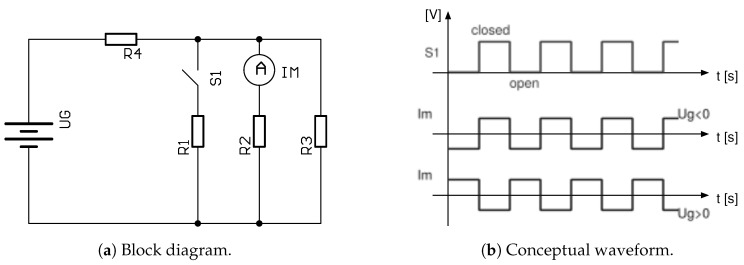
Basic principle of constant (DC) magnetic flux measurement.

With this in mind, an active element such as a transistor is required, which would allow the fluxes inside the magnetic circuit to be redirected. Having achieved that, the resulting flux changes can be easily captured with a winding. The working principle would then be as shown in Figure 3. The $U_{G}$ generates magnetic flux via a “primary” path denoted by the $R_{4}$ to the newly designed flux sensor represented by three branches, the resistances $R_{1}$ and $R_{2}$, plus some of the leaked by flux, which is depicted by $R_{3}$. By altering the path of $R_{1}$, the flux through $R_{1}$ changes, as it does through $R_{2}$ and $R_{3}$. The resulting change of flux $I_{m}$ can be observed as induced voltage $N\frac{\delta \phi }{\delta t}$ flowing through $R_{2}$. If the polarity of the incoming current and thus the flux changes, so does the output phase, as depicted by $I_{m}$ in the chart.

The challenge therefore lies in creating a new basic element $S_{1}$ for magnetic circuits with a current controlled variable reluctance (CCVR). To avoid any ambiguity, it is important to note that variable reluctance has up to date referred to motors and sensors, where the reluctance changes due to the mechanical movement of a part of a magnetic circuit, which is not the case with CCVR, where the reluctance is controlled by an electric current.

### 3.2. Current Controlled Variable Reluctance

**Figure 4:**
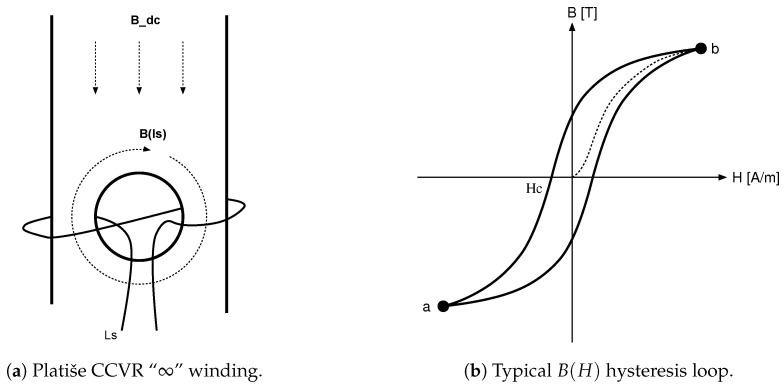
Current controlled variable reluctance.

Soft-magnetic materials B(H) have non-linear characteristics and saturation properties, as shown in Figure 4b, which is why an externally driven current may induce magnetic force and drive the core into the region of saturation, close to or into Points (a) or (b). In these two final saturated points, the relative permeability is reduced down to $\mu_{r}\to 1$.

Figure 4a illustrates the implementation of the CCVR with an opening section in a magnetic material, creating a self-closed magnetic loop $B(I_{s})$ that is excited by a balanced winding $L_{s}$ on both branches of the opening section. Such a winding $L_{s}$ has an infinity form, which may be also wound as two independent windings. By adding an electric current into $L_{s}$, a portion of the magnetic material is actively being pushed closer to the saturation points. Let us assume that the external field of interest $B_{dc}$ is flowing downwards, as depicted in Figure 4a. The field $B(I_{s})$ is subtracting from the $B_{dc}$ on the left side, while it is adding on the right, approaching the saturation region as a sum $B\left(I_{s}\right)+B_{dc}$ first. Whenever either part nears the saturation region, the effective magnetic resistance reduces, and if $B\left(I_{s}\right)\gg B_{dc}$, the entire region is affected quasi homogeneously.

Figure 3 proposes a switching operation of the CCVR using $S_{1}$. A balanced structure ensures that $B(I_{s})$ does not leak away significantly and interfere with the $B_{dc}$, i.e., the flux of our interest induced by some primary current. Reducing the leakage also reduces noise injection to the primary and compensation winding of the transducer.

### 3.3. Complete Sensor

**Figure 5:**
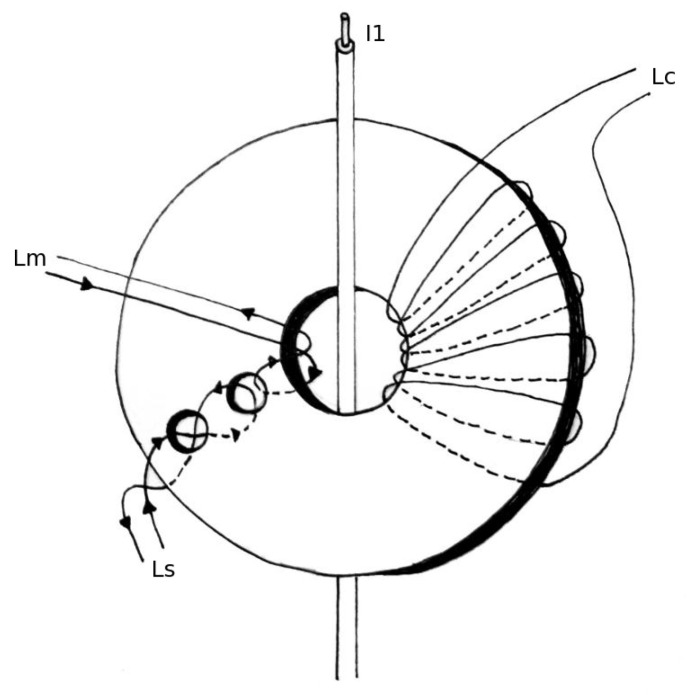
DC-CT core with Platiše Flux Sensor, primary and compensation winding.

Figure 5 illustrates the conceptual implementation of the Platiše Flux Sensor on a single gapless toroid core. In this implementation, an additional opening was made to create the path $R_{1}$ as per Figure 3, with an A-meter $I_{m}$ as an additional winding $L_{m}$. To make the core ready for the transducer described in Section 4, a single turn $N_{1}$ for the primary current $I_{1}$ is added, as well as a secondary winding of $N_{c}$ turns for the compensation current $L_{c}$.

**Figure 6:**
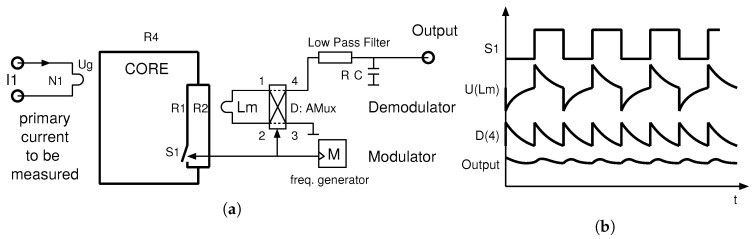
Concept of the Platiše Flux Sensor DC measurement. (**a**) Block diagram; (**b**) Waveform diagram.

Figure 6a shows a block diagram of the minimum required control circuitry for the current transducer, consisting of:

- *M*, a modulator in the form of a simple digital clock generator of a duty cycle 50%:50%,
- *D*, an analogue multiplexer AMux as demodulator synchronized to the modulator, in which two states with pairs (1–4, 2–3) and (1–3, 2–4) operate as a ±1 sign mixer,
- a low pass filter used to remove glitches and transients sensed by $L_{m}$ and demodulated by *D*.

The operation of the circuit is shown in Figure 6b; it has a conceptual waveform with an output voltage directly proportional to the current of interest $I_{1}$. For simplicity, let us consider a primary current $I_{1}$ is generating a constant flux in the core. The CCVR ($S_{1}$) then redistributes this constant flux among paths $R_{1}$ and $R_{2}$ so that its changes get captured by $L_{m}$ and demodulated. $R_{1}$ and $R_{2}$ are typically significantly smaller than $R_{4}$, and since $R_{2}$ does not change, the alteration of the resistance in the $R_{1}$ path does not significantly impact the flux magnitude changes in $R_{4}$. A low-noise operation is ensured by the parallel branch $R_{2}$, the closed magnetic loop path of the CCVR in $R_{1}$, and a bandwidth-controlled variable reluctance control current driver. Changing the direction (sign) of $I_{1}$ produces a 180 degree phase shifted $U(L_{m})$, but of an equal signal pattern, and the resulting output at D(4) is rectified as negative in the amplitude. The sensor bandwidth is defined by the modulator and demodulator sampling frequency, which may in practice go up to 500 kHz or more.

## 4. Direct Current-Current Transducer

The output of the Platiše Flux Sensor may be treated in exactly the same way as the output of a Hall sensor, a magneto-resistance sensor, or a flux-gate sensor, with one significant difference: the Platiše Flux Sensor does not have an air-gap, which allows the complete magnetic circuit to reach an effective permeability $\mu_{e}$ as high as 10 k for soft-magnetic cores, 100 k for nano-crystalline cores, and even higher for special composite materials. This superior magnetic amplification is directly comparable to zero-flux sensors and the simplicity of implementation is comparable to standard flux-gate or Hall-based sensors.

**Figure 7:**
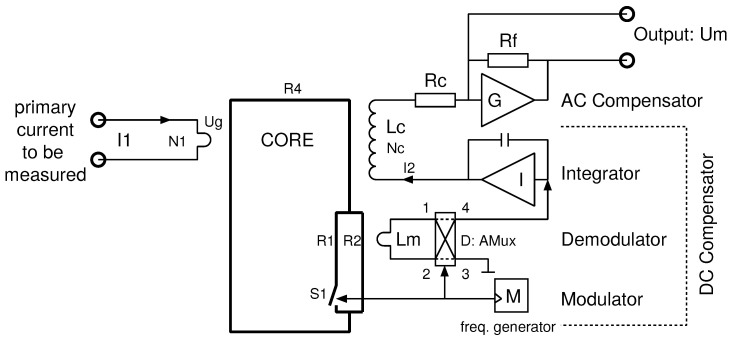
Block diagram of the DC-CT with the Platiše Flux Sensor.

Furthermore, high-permeability materials have very low coercivity forces ($H_{c}$), but are temperature dependent and have non-linear B(H). For this reason, the best use of a Platiše Flux Sensor for current sensing applications is in a closed-loop null-method; the same as applies to Hall sensors, flux-gate sensors, and magneto-resistance closed-loop current transducers. A block diagram of this implementation is shown in Figure 7. In reference to Figure 6a, this block diagram adds:

- a compensation winding $L_{c}$,
- an integrator *I* with a feedback amplifier for driving the compensation winding $L_{c}$, and
- an optional trans-impedance amplifier *G* used to convert current into voltage via a low-impedance termination. It also decreases the cut-off frequency of the AC path and thus increases both the stability and the AC performance of a sensor.

The simplified transfer function of the transducer can be derived from two parts, first the direct AC path through the transformer and second the DC compensation part. By neglecting the influence of the primary winding and compensation winding stray inductances and capacitances, the properties of the soft-magnetic material, and the almost zero-ohm input resistance of the trans-impedance amplifier *G*, the secondary current $I_{2}$ is:

(4) $$I_{2}=I_{1}\frac{N_{1}}{N_{c}}\cdot \frac{sL_{c}/R_{c}}{1+sL_{c}/R_{c}}+\frac{V_{i}}{R_{c}+sL_{c}}$$

where $I_{1}$ is the primary current of $N_{1}$ turns, the secondary winding of $N_{c}$ turns, inductance $L_{c}$, and series resistance $R_{c}$. The $V_{i}$ is the output voltage from the feedback amplifier *I*. Typically, the primary conductor has a single turn; however, more turns scale down the input current range, as well as the magnetic offset and hysteresis. To simplify the model, $N_{1}=1$ is used in the following. Figure 7 in addition provides trans-impedance amplifier *G*, which converts and amplifies the $I_{2}$ current to a voltage $U_{m}$ through the resistor $R_{f}$, i.e., $U_{m}=I_{2}\cdot R_{f}$.

Due to the gapless core construction, the sensitivity of the sensor and inductance $L_{c}$ is very high, as well as the time constant $\tau_{c}=L_{c}/R_{c}$ with cut-off frequencies below 10 Hz. This means the required gain of a Platiše Flux Sensor is low, although its sampling frequency is very high, making the DC-CT easily stable and producing low noise. In such a transducer, the wide bandwidth AC part of the $I_{1}$ flows directly through the core and the compensation winding $L_{c}$, while the output from the flux sensor compensates for the small errors starting at a few hundreds Hz down to DC. The ideal resulting current through the $L_{c}$ is thus $N_{1}/N_{c}$.

The DC compensation part as represented in Figure 7 with the additional amplifier *I* offers bi-directional operation from a single supply. For stable operation, it also requires a proportional part of the regulation loop to be able to guarantee a sufficient phase margin for stability. Therefore:

(5) $$V_{i}=V_{p}\left(K_{p}+\frac{K_{i}}{s}\right)$$

where $K_{p}$ and $K_{i}$ are PI regulator parameters and $V_{p}$ is the residual flux in voltage as demodulated by the Platiše Flux Sensor. It can be simplified to a simple linear relation around the zero and within the limited range of approximately ±250 mA, to represent the difference between the primary and compensated currents:

(6) $$V_{p}=S\cdot \left(I_{1}-N_{c}I_{2}\right)$$

where *S* is the sensitivity of the CCVR in V/A referred to input (RTI). Substituting it all together yields the basic input/output transform function of the DC-CT:

(7) $$I_{2}=\frac{I_{1}}{N_{c}}\cdot \frac{s^{2}Lc+sK_{p}N_{c}S+K_{i}N_{c}S}{s^{2}L_{c}+s\left(R_{c}+K_{p}N_{c}S\right)+K_{i}N_{c}S}$$

Before the actual implementation of the proposed DC-CT as a functional prototype, the proposed concept, assuming an entirely new structure of the core with multiple openings, was verified with the powerful, free, and open source ngspice simulator (http://ngspice.org), to minimize the probability of unnecessary work and expensive trial and error iterations. To this end, the ngspice repository was forked and upgraded to a comprehensive open source system simulation suite that includes the analog, digital, Verilog, and firmware of the digital controller written in C (http://isotel.org/mixedsim). This allowed a comprehensive evaluation and simulation of the design variants of the control loop, which in themselves are out of the scope of this article.

**Figure 8:**
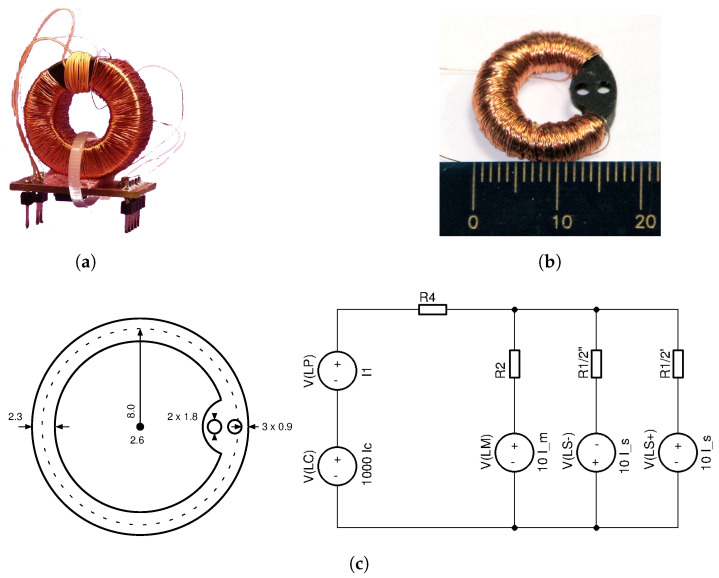
Soft ferrite core prototypes. (**a**) The first proof-of-concept ±40 A DC-CT; (**b**) The first custom made toroid DC-CT core 40A9R; (**c**) Drawing in mm with simplified schematics of the core 40A9R.

Following the initial successful simulation runs, the first proof-of-concept of a ±40 A current transducer, shown in Figure 8a, was implemented by using an existing off-the-shelf soft-ferrite core. The analog electronics implementation in a programmable chip on the PCB below the core followed the scheme, as shown in Figure 7. This first proof-of-concept DC-CT was thoroughly tested before developing the first custom designed core 40A9R rated for ±40 A along with the technology processes for industrialization of such core designs. The 40A9R core with the secondary winding is shown in Figure 8b, the drawing and simplified schematics in Figure 8c, and the key core parameters listed in Table 1.

## 5. Testing and Performance Evaluation

The testing and performance evaluation of the proposed DC current transducer based on the novel Platiše Flux Sensor was carried out in a purposely built development and automated test environment (ATE). To this end, a high-current digital linear low-noise DC/AC amplifier was developed that can handle up to 40 A at DC, 25 A up to 100 kHz, 4 A at 1 MHz, and a maximum bandwidth of 2 MHz. Multiple turns were used to achieve higher ampere-turns. To ease the firmware development, testing, and integration with ATE systems, the ISOTEL Device Manager (IDM) software was used (http://isotel.org/idm), freely available for download, together with the Python/Jupyter integration (https://jupyter.org). Further equipment used in the tests was comprised of a MACC${}^{plus}$ 600 A with a Vishay VFR S-series 2 ppm/K reference shunt resistor (http://www.vishaypg.com/docs/63001/63001.pdf), a modular power system HP66000A, a digital multimeter HP34401A, a data acquisition system Dewesoft Sirius for <100 kHz measurements, a digital oscilloscope R&S RTO1004 for all other measurements, various temperature chambers, and supporting amplifiers, power supplies, and other accessories.

The following sub-sections present the key performance measurements obtained for the first DC-CT prototype with the custom designed core 40A9R.

### 5.1. The Platiše Flux Sensor Operating Characteristics

**Figure 9:**
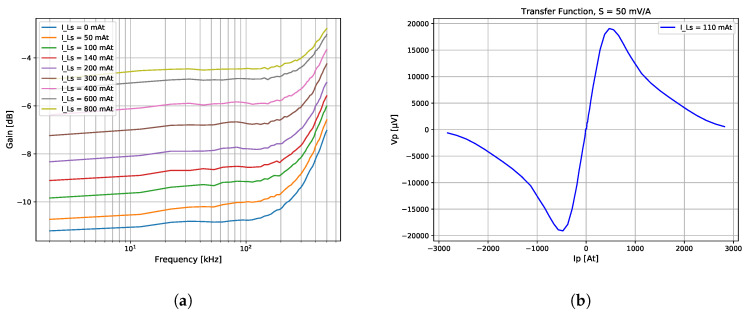
Platiše Flux Sensor characteristics of the 40A9R. (**a**) CCVR characteristics; (**b**) Sensor transfer function.

Referring to Section 3.2, the key principle of the Platiše Flux Sensor is the flux redirection inside the core. With the presence of the control current through the winding $L_{s}$, path $R_{1}$ closes, thus redirecting the magnetic flux through path $R_{2}$. The measurement results are shown in Figure 9a as a function of frequency through the primary winding and DC through $L_{s}$, taking amplitude measurements on $L_{m}$.

The overall sensitivity and operational range of the core is shown in Figure 9b as a function of output rectified, but not amplified voltage $V_{p}$ and input current $I_{p}$, at the given peak-to-peak $L_{s}$ current of 110 mAt. For this particular core 40A9R, the current of 1 A converts to 50 mV of demodulated voltage output with the operational range of ±0.5 A; therefore, $S=50\;\frac{\mathrm{mV}}{A}$ as per Equation (6). Beyond this range, the material around the two openings begins entering the saturation region, and the signal begins to fall.

### 5.2. DC Linearity and Accuracy

**Figure 10:**
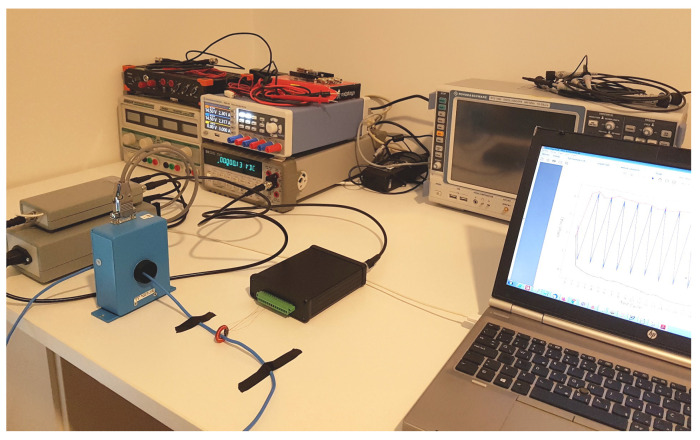
Test bench with 40A9R connected to the DC-CT evaluation controller, MACCplus 600A, HP334401A, RTO1004, NGE100, and HP66000A with the custom developed 2 MHz generator under the table.

In order to evaluate the linearity and accuracy of the prototype DC-CT, we used the testing setup depicted in Figure 10 with:

- HP66000A as a current source,
- MACCplus 600A as a reference instrument,
- Vishay VFR S-Series, S102K (50.000 Ω, 0.01%, 4.5 ppm/K) and S102CT (100.00 Ω, 0.01%, 3.5 ppm/K) in parallel, as a burden resistor of value 33.333 Ω, and
- HP34401A as the 6.5 digit voltage meter.

The three major contributors of DC errors all relate to the core material:

- The initial offset due to the magnetization of the core $H_{c}$; this can to a large extent be improved using the degauss procedure of the form $A\sin\left(\omega t\right)\cdot e^{-t/T}$ to reset the core to its initial condition.
- The hysteresis of the core material, as a result of a non-homogeneous distribution of the magnetic fields between the primary conductor and the core in a zero-flux configuration.
- The temperature drift, which is directly proportional to the remaining offset in the magnetic core due to the previous two error sources and the temperature-related changes of the material itself.

The modulation and demodulation principles in a Platiše Flux Sensor are designed to cancel all electric offsets that would have come from the flux sensor itself. The major electric error is thus introduced by the trans-impedance amplifier attached to the secondary compensation winding. In this design, a long-term stable resistor with 0.2 ppm/K from the Vishay Z-Foil VSMP series 1206 (http://www.vishaypg.com/docs/63060/VSMP.pdf) was used, which defines the gain stability. The measurement uncertainties of the entire setup are given in Table 2.

**Figure 11:**
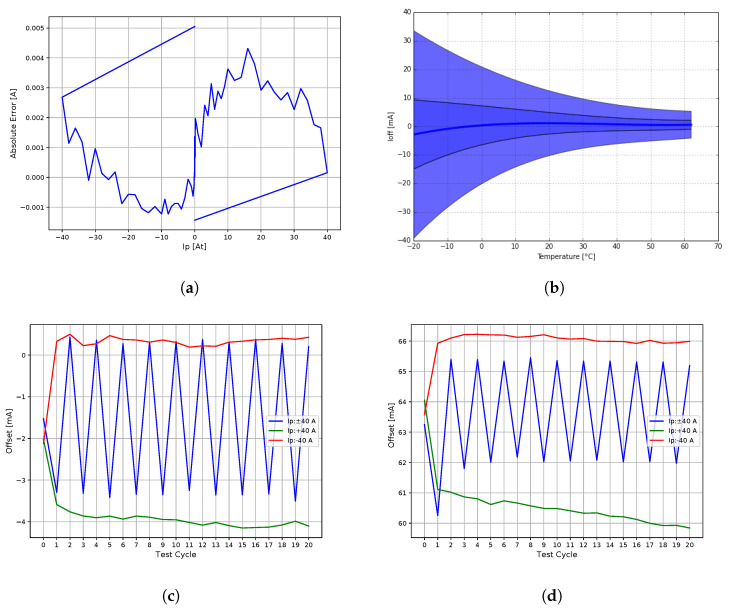
Accuracy of the 40A9R core. (**a**) Hysteresis and linearity; (**b**) Temperature stability at 25 A; (**c**) Cycling at the rated range after degaussing is applied; (**d**) Cycling at the rated range without degaussing.

Figure 11a illustrates the linearity and hysteresis loop of the corrected gain, calculated as the best fit straight line (BFSL) between the primary and secondary currents. The measurement was taken after applying degaussing in each direction to the rated range (*X* axis), while the *Y* axis represents the absolute error from the BFSL. Hysteresis changes and drifts versus series of cycles from zero to positive and negative maximum rated range, and alternating between the minimum and maximum, are shown in Figure 11c after the degaussing is applied, and Figure 11d is without the degaussing.

The closed-loop operation ensures a very high linearity, with a peak-to-peak of 6 mA within the entire 80 A range. The gain is slightly affected by the primary winding position and the asymmetrical winding due to additional openings in the core, which was to some extent elaborated in [17]. As the linearity is about 200 ppm at k=2 and by taking into account the stability part of the VFR uncertainty only (<45 ppm), gain accuracy specification was adopted from the Vishay VSMP burden resistor specifications. The worst case measurements are summarized in Table 3.

Temperature stability at a continuous and constant current of 25 A applied after the degauss procedure was measured on 11 samples in a temperature chamber; the results are shown in Figure 11b. The outer envelope represents ±3σ, the inner ±σ the standard deviation interpolated with a polynomial, and the curve in the center the mean value.

### 5.3. Noise

Noise was evaluated by using the RTO1004 oscilloscope and direct connection of the core 40A9R to a burden resistor of 8 Ω; system noise floor accounted for: 83 μ$A_{\mathrm{rms}}$ @ 10 kHz, 283 μ$A_{\mathrm{rms}}$ @ 100 kHz, and 972 μ$A_{\mathrm{rms}}$ @ 1 MHz bandwidth.

The operation of the proposed DC-CT identified three major sources of noise:

1. periodic excitation used in CCVR, which is seen as a ripple at a constant frequency and its harmonics, above 100 kHz.
2. a compensation regulation loop with the amplitude depending on the sensitivity of the sensor and loop parameters, below 10 kHz.
3. output electric noise.

Table 3 lists the noise of the 40A9R core with the CCVR driven by a purely digital output driver operating at 220 kHz measured on the Vishay VSMP shunt resistor of 33.2 Ω and a bandwidth-controlled (limited) voltage input.

### 5.4. AC Performance

The practical AC bandwidth limits of the DC-CT are defined by the core material, the electronics circuitry, and the wiring methods of the compensation winding, as well as the complete primary winding circuit. Note that one meter of a straight wire of 4 mm in diameter already has a self-inductance of 1.2 μH and a resistance of 1.4 mΩ. The time constant τ=L/R is nearly 1 ms and limits the excitation rise times. For this reason, the primary loop needs to be minimized and the wire twisted in the test setup. A non-ideal compensation winding includes the parasitic capacitance between the windings, to the core, and leakage inductance that distorts the flatness and may show up as resonances at certain frequencies. In this setup, we used:

- a custom made 2 MHz generator with integrated RedPitaya (https://www.redpitaya.com/) as the DAQ and
- the RTO1004 oscilloscope.

The Bode diagram was calculated by cross-correlating the reference input current with the output of the DC-CT. Flatness accuracy was not evaluated specifically in this case.

**Figure 12:**
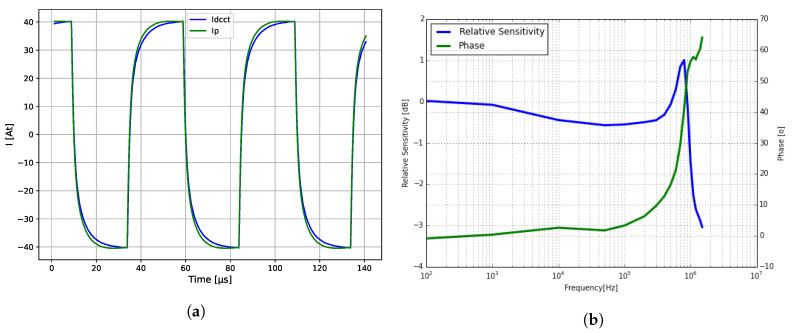
AC response of the 40A9R. (**a**) Large step response of the 40A9R core; (**b**) Small signal bandwidth, 15% *I_p_* until 100 kHz, and 3% *I_p_* at 1 MHz.

Figure 12a shows large signal step responses and Figure 12b the bandwidth of the 40A9R transducer. During this particular test, the AC current at 100 kHz was over 6 A, gradually decreasing to about 1.5 A at 1 MHz.

### 5.5. Discussion

Table 3 compares the results obtained for the DC-CT prototype based on the Platiše Flux Sensor with the custom designed core 40A9R and those obtained for the selected best available sensors from LEM using different methods and technologies. The proposed Platiše Flux Sensor outperforms other technologies in many aspects. The power consumption is slightly higher compared to the closed loop LKSR and LZSR due to the lower turn ratio, to achieve wider bandwidth performance. The key advantage of the three-core flux-gate technology, as shown in Figure 2, is the low offset that comes at the price of higher power consumption, size, and cost.

At the time of writing, we improved the total offset of the Platiše Flux Sensor by using higher permeability materials without the required degaussing to a typical value of approximately 15 mA (reduced from 27 mA, achieved with 40A9R) and the worst below 50 mA (reduced from 90 mA, achieved with 40A9R); the gain of the CCVR was improved by an additional 20 dB at the same excitation current $I_{Ls}$.

After the first prototype core 40A9R, the second version of the core was produced in a series of about 100 pieces with more or less equal characteristics. These cores were also extensively tested by Elettra Sincrotrone Trieste and used with the degauss procedure in their development of a ±20 A, a 15 ppm long-term stable power supply for the European Spallation Source [18].

## 6. Conclusions

This article presents a novel type of magnetic flux sensor named after the inventor Platiše, proposes its use in a DC current transducer, and provides the results of a thorough testing and performance evaluation of the first DC-CT prototype. The gapless core construction and the use of the closed-loop null method with a compensation winding yield a superior performance compared to other widely used DC current sensors based on different methods, while also promising a simple and low-cost implementation. It enables compact and rigid DC current sensors that are also able to address automotive and other critical application areas in addition to the high-performance and high-current measurement devices for metering, testing, and measurement. In fact, significant technology improvements, which have become available in recent years, such as high-permeability temperature-stable magnetic materials and low-cost silicons with increased computing power, have enabled the prototyping and product industrialization of the concept that was initiated over a decade ago.

The initial DC-CT prototype based on the custom designed toroid core 40A9R presented in this article achieved very high linearity below 200 ppm for a 40 A DC rated transducer, a sensitivity of over 0.5 mA, a bandwidth of over 800 kHz, very low noise of 0.5 mArms below 100 kHz, and 1.6 mArms up to MHz approaching the performances of costly, power hungry, and bigger three-core flux-gate devices.

Recently, we developed higher performance DC-CT control electronics suitable for the evaluation of cores over 1000 A and a bandwidth over 10 MHz and used it to repeat and validate the measurements presented in this article.

Further product development includes in particular industrialization of the novel core designs, the designs of precision higher current cores, and the test systems with the aim to meet compliance with corresponding general (e.g., IEC 61869-14:2018 for instrument transformers) and domain-specific standards, and specific customer requirements.

## 7. Patents and Trademarks

Extensive patent search has been performed by a third party in Germany to validate the novelty of the Platiše Flux Sensor and the overall DC-CT solution with this new sensor. The innovation was filed as PCT/EP2013/003278, and by the time of the submission of this paper, it already was granted in US9927464B2, JP6305419B2, MX342727B, CN104871014B, and India 336089; the applications in progress include EP2914967A1, WO2014067660A1, DE102012021364A1, and BR112015009522A2.


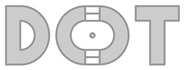


The DC-CT trademark was registered under the EUIPO filing number 015057102, in which the center of the design is made out of two C cores, forming a toroid core, attached to each other via the two openings, representing the Platiše Flux Sensor. The dot in the center represents the primary conductor. The two C-cores represent the “CC” letters of the DC-CT keyword even though it may also read as “DOT”. DC-CT official web page is located at https://www.dc-ct.com.

## Figures and Tables

**Table 1:** Structural and electrical parameters of the 40A9R core.

Parameter	Value	Unit
Diameter	18.3	mm
Thickness	2.6	mm
Width	2.3	mm
Cross-sectional area $R_{1},R_{2}$	2	mm${}^{2}$
Cross-sectional area $R_{4}$	6	mm${}^{2}$
Effective length $R_{1}$ of $L_{s}$	10	mm
Effective length $R_{2}$ of $L_{m}$	6	mm
Effective length $R_{4}$ of $L_{c}$	40	mm
Material	26G by Iskra Feriti	-
Relative Permeability $\mu_{r}$	2200	-
Coercivity $H_{c}$	12	A/m
Sensor winding $L_{s}$	20|560	turns|uH @ 100 kHz
Sensor winding $L_{m}$	10|254	turns|uH @ 100 kHz
Compensation winding $L_{c}$	1000|23|0.71	turns|Ω|H @ 10 kHz

**Table 2:** Measurement uncertainty evaluated at 10 and 40 A of primary current, and k=1.

Standard Measurement Uncertainty	$u_{c}$ @ 10 A (ppm)	$u_{c}$ @ 40 A (ppm)
40A9R current reading (Type A, N = 10)	8	2
HP34401A as 6.5 digit volt meter from specifications and 90 days	98	34
MACCplus 600 A from specs with 10 ${}^{\circ }$C temperature rise and 90 days	13	13
Uncorrected Vishay VFR S-series resistor from specs with 10 ${}^{\circ }$C rise	84	84
**Uncorrelated sum**	**130**	**92**

**Table 3:** Performance comparison of similar current transducers and different technologies.

	DC-CT 40A9R	LEM IT 60A ${}^{1}$	LKSR-50NP ${}^{2}$	LZSR-80P ${}^{3}$
Technology	Platiše-Flux	3-Flux-Gate	S-Flux-Gate	Closed-Hall
Turn Ratio	1000	600	1600	2026
Rated Primary Current $I_{p}$ (A)	40	60	50	80
Linearity (ppm)	<200	20	1000	-
Voltage Out Gain Accuracy (%)	0.1	-	0.8	0.8
Voltage Out Gain Drift (ppm/K)	10	-	40	75
Total Worst Offset RTI (mA)	±27	±15	±140	±414
...without degauss and starting at $I_{p}\neq 0$	±90	-	-	-
Offset Drift RTI (uA/K)	50	150	<700	240
Step Response to 90% (us)	1	1	0.4	>3
Bandwidth at 0.5% $I_{p}$, −3 dB (kHz)	>800	800	300	200
Reaction time, 0 to 10% of $I_{p}$ (μs)	0.1 @ 30 $\frac{A}{\mu s}$	-	0.3 @ 50 $\frac{A}{\mu s}$	1 @ 50 $\frac{A}{\mu s}$
Time Response, 0 to 90% of $I_{p}$ (μs)	1.5 @ ≈15 $\frac{A}{\mu s}$	1 @ 25 $\frac{A}{\mu s}$	0.4 @ 50 $\frac{A}{\mu s}$	≈3 @ 50 $\frac{A}{\mu s}$
Noise RTI < 10 kHz (mArms) ${}^{4}$	0.2	0.4	29	6
Noise RTI < 100 kHz (mArms) ${}^{4}$	0.5	0.9	38	10
Noise RTI < 1 MHz (mArms) ${}^{4}$	1.6	-	58	29
Power Min …Max (W)	0.13 …0.33	2.4 …3.9	0.1 …0.24	0.1 …0.2

${}^{1}$ LEM IT 60, https://www.lem.com/sites/default/files/products_datasheets/it_60-s_ultrastab.pdf; ${}^{2}$ LKSR, https://www.lem.com/sites/default/files/products_datasheets/lksr_series.pdf; ${}^{3}$ LZSR-80P, https://www.lem.com/sites/default/files/products_datasheets/lzsr_80-p.pdf; ${}^{4}$ the RMS noise of LEM products was calculated as peak-to-peak/6.6.

## Abbreviations

The following abbreviations are used in this manuscript:

40A9R	The first prototype, ±40 A DC-CT toroid core
BFSL	Best fit straight line
CCR	Current controlled reluctance
CCVR	Current controlled variable reluctance
DC-CT	Direct current-current transducer (transformer)
IDM	ISOTEL Device Manager
RTI	Referred to input
